# In Vitro Bioaccessibility of Extractable Compounds from Tannat Grape Skin Possessing Health Promoting Properties with Potential to Reduce the Risk of Diabetes

**DOI:** 10.3390/foods9111575

**Published:** 2020-10-30

**Authors:** Adriana Maite Fernández-Fernández, Amaia Iriondo-DeHond, Tiziana Nardin, Roberto Larcher, Eduardo Dellacassa, Alejandra Medrano-Fernandez, María Dolores del Castillo

**Affiliations:** 1 Departamento de Ciencia y Tecnología de Alimentos, Facultad de Química, Universidad de la República, General Flores 2124, Montevideo 11800, Uruguay; afernandez@fq.edu.uy (A.M.F.-F.); amedrano@fq.edu.uy (A.M.-F.); 2 Instituto de Investigación en Ciencias de la Alimentación (CIAL) (CSIC-UAM), C/ Nicolás Cabrera, 9, Campus de la Universidad Autónoma de Madrid, 28049 Madrid, Spain; amaia.iriondo@csic.es; 3 Graduate Program in Chemistry, Facultad de Química, Universidad de la República, General Flores 2124, Montevideo 11800, Uruguay; 4 Dipartimento Alimenti e Trasformazione, Centro Trasferimento Tecnologico, Fondazione Edmund Mach di San Michele all’Adige, Via E. Mach, 1 38010 S. Michele all’Adige (TN), Italy; tiziana.nardin@fmach.it (T.N.); roberto.larcher@fmach.it (R.L.); 5 Departamento de Química Orgánica, Facultad de Química, Universidad de la República, General Flores 2124, Montevideo 11800, Uruguay; edellac@fq.edu.uy

**Keywords:** anti-inflammatory, antioxidant, α-amylase, bioaccessibility, diabetes, α-glucosidase, glucose transporters, Tannat grape skin extract

## Abstract

Diabetes pathogenesis encompasses oxidative stress, inflammation, insulin malfunctioning and partial or total insulin secretion impairment, which leads to a constant hyperglycemia. Polyphenols are known to possess bioactive properties, being Tannat grape skin a natural and sustainable source of these compounds. The present study aimed to find out the bioaccessibility of health-promoting molecules composing a multifunctional extract from Tannat grape skin obtained under hydro-alcoholic-acid conditions. The identification of phenolic compounds in the samples was performed by ultra-high performance liquid chromatography tandem mass spectrometry (UHPLC-MS/MS). Subsequently, the samples were in vitro digested mimicking the human oral gastrointestinal conditions and the bioactivity of the digest (antioxidant, anti-inflammatory and modulation of glucose metabolism) was assessed. Effect on glucose metabolism was estimated by measuring carbohydrases activity and the functionality of glucose transporters of small intestine cells in presence and absence of the digested extract. Flavonoids, phenolic acids and phenolic alcohols were the major phenol compounds detected in the extract. The bioaccessible compounds protected the intestinal cells and macrophages against the induced formation of reactive oxygen species (ROS) and nitric oxide (NO). In addition, glucose transporters were inhibited by the digested extract. In conclusion, the bioaccessible compounds of the extract, including phenols, modulated key biochemical events involved in the pathogenesis of diabetes such as oxidative stress, inflammation and glucose absorption. The extract was effective under prevention with co-administration conditions supporting its potential for either reducing the risk or treating this disease.

## 1. Introduction

Tannat grape skin is characterized by its full anthocyanins profile at maturity [1]. They can be bound to polysaccharides at the cell wall through hydrogen bonds and hydrophobic interactions, confined in vacuoles or associated with the nucleus in plant cells [2], making necessary different extraction conditions for their release. During red winemaking, anthocyanins still remain in high amount at the pomace due to skin matrix retention [2]. Phenolic compounds of Tannat grape skin have been associated to antioxidant, antidiabetic, antiobesity, and anti-inflammatory properties [3].

The pathogenesis of diabetes involves chronic inflammation associated with nitric oxide (NO) and reactive oxygen species (ROS) production by macrophages, increased ROS blood levels, partial or full insulin secretion impairment in the pancreas, and insulin resistance through insulin receptor impairment [4,5] with the subsequent constant hyperglycemia. Consequently, a global approach is needed for its prevention/treatment. ROS, pro-inflammatory cytokines and nuclear factor κB (NF-κB) transcription factor are involved in the inflammatory process [4]. The reduction of their formation may be helpful for reducing the risk of diabetes. In addition, the modulation of glucose metabolism by inhibition of carbohydrases activity and glucose transporters is effective for a good control of blood glucose levels [6]. Therefore, natural sources of bioactive compounds able to regulate glucose metabolism and possessing antioxidant and anti-inflammatory properties are of great interest for reducing the risk and treating diabetes.

The present study pretended to provide novel information on the chemical composition of the polyphenol fraction of the extract which may play a fundamental role on events associated with the pathogenesis and clinical symptoms of diabetes. Although the Tannat grape skin hydro-alcoholic-acid extract seems to be a good candidate for the prevention/treatment of diabetes, the bioaccessibility of its compounds providing these properties and their effect on glucose transport have not been previously studied. Consequently, the aim of the present work was to contribute to this fundamental knowledge.

## 2. Materials and Methods

### 2.1. Materials

Tannat grape pomace was provided by Bouza wine cellar (Montevideo, Uruguay). All the chemicals were of reagent grade. Cyanidin chloride standard (Cyd) was from Chengdu Biopurify Phytochemicals Ltd. Reagents for total carbohydrates content were from Sigma-Aldrich (St. Louis, MO, USA). Folin reagent, 2,20-azinobis-(3-ethylbenzothiazoline-6-sulfonic acid) diammonium salt (ABTS), 6-hydroxy-2,5,7,8-tetramethylch-roman-2-acid (Trolox), fluorescein (FL) disodium salt, 2,20-azobis (2-methylpropionamidine) dihydrochloride (AAPH), α-glucosidase (rat intestine acetone powder), acarbose, 4-methylumbelliferyl-α-D-glucopyranoside, α-amylase from human saliva (Type IX-A, lyophilized powder, 1000–3000 units/mg protein), starch, maltose standard, 3,5-dinitrosalicylic acid, and dimethyl sulfoxide (DMSO) were purchased from Sigma-Aldrich (St. Louis, MO, USA). 

For cellular studies, Dulbecco’s modified Eagle medium (DMEM), l-Glutamine, antibiotics (penicillin and streptomycin) and trypsin were from Gibco Laboratory (Invitrogen Co, Grand Island, NY, USA), while fetal bovine serum (FBS) was from Hyclone (GE Healthcare, Chicago, IL, USA). In addition, 3-(4,5-dimethylthiazol-2-yl)-2,5-diphenyltetrazolium bromine (MTT), 2′.7′-dichlorofluorescin diacetate (DCFH-DA) and ascorbic acid were obtained from Sigma-Aldrich (St. Louis, MO, USA). Sulfanilamide, N-(1-napthyl) ethylenediamine dihydrochloride, phosphoric acid, sodium nitrite and lipopolysaccharide from E. coli O55:B5 (LPS) were also purchased in Sigma-Aldrich (St. Louis, MO, USA). Glucose transport inhibition standards (Phloretin ≥ 99% and phloridzin dihydrate 99%) were purchased from Sigma -Aldrich (St. Louis, MO, USA).

### 2.2. Methods

#### 2.2.1. Grape Pomace Treatment and Extract Preparation

Seeds and skins of Tannat grape pomace were manually separated and the skin was submitted to drying at 40 °C up to constant weight, achieved at 24 h [3]. Skin was then powdered by employing a domestic mill.

Extract was prepared by weighting 20 g of Tannat dried grape skin powder and adding 200 mL of solvent (methanol-water-formic acid, 70:25:5) (hydro-alcoholic-acid extraction, EHAA). The extract (EHAA) was then filtered through filter paper (Whatman n°4), the supernatant dried in a rotary evaporator, then diluted with distilled water, freeze dried for 4 days, and stored at −20 °C for subsequent analysis.

#### 2.2.2. Mass Spectrometry Analysis

Extract solution (10 mg/mL in H_2_O-MeOH, 50:50, *v*/*v*) was analyzed by UHPLC-MS/MS using a Thermo Ultimate R3000 UHPLC (Thermo Scientific, Sunnyvale, CA, USA) for chromatographic separation, equipped with a Rheodyne 6-port automated switching valve used for on-line clean-up, adopting the method recently proposed by Barnaba et al. [7]. Identification of phenolic compounds was performed using a QExactive TM hybrid quadrupole-orbitrap mass spectrometer (HQ-OMS, Thermo Scientific, Bremen, Germany) equipped with heated electrospray ionization (HESI-II). Mass spectra were acquired in negative ion mode through full MS-data dependent MS/MS analysis (full MS-dd MS/MS), recording full mass spectra at a mass resolving power of 140,000 full width at half-maximum (FWHM), and data-dependent mass spectra at 17,500 FWHM. The mass spectrometer experimental conditions were those previously reported [7]. Full mass spectral data were used for identification and quantification of individual phenolic compounds. Their presence in the samples was identified through data-dependent mass spectral results, by matching MS/MS spectra with those obtained from previous experiments performed on standard solutions and collected as a spectral library in the Thermo Library Manager Application (Thermo Scientific, San Jose, CA, USA).

#### 2.2.3. Bioaccessibility of Bioactive Compounds

##### Release of Bioaccessible Compounds

In vitro oral-gastro-intestinal digestion was performed according to Hollebeeck et al. [8]. The digests obtained were fractionated (centrifuged at 10,000 rpm for 10 min) and the soluble fraction containing the bioaccessible compounds was subjected to a clean-up prior to analysis. Cholestyramine resin was used for taking bile out of the mix so the bioaccessible fractions could be tested on cell culture. Briefly, the supernatant was stirred with 10% *w*/*v* of resin for one hour at room temperature. Then, it was centrifuged at 5000 rpm for 15 min and the supernatant was filtered. Soluble fractions were frozen and lyophilized for further analysis.

##### Antioxidant Assays

- In vitro antioxidant capacity studies

The overall antioxidant properties of the bioaccessible compounds were evaluated by analysis of the total polyphenol content (TPC), ABTS and ORAC-FL assays.

TPC was performed by the Folin-Ciocalteau method [9] with modifications described by Fernández-Fernández et al. [3] using a gallic acid standard curve ranged from 0.05 to 1.0 mg/mL. Results were expressed as mg GAE/g of dry sample.

ABTS method [10] with modifications [3] was performed to determine electron transfer (ET) capacity using a trolox calibration curve (0.25 to 1.5 mM). Hydrogen atom transfer (HAT) capacity was performed by ORAC-FL assay [11] with some modifications [3]. Trolox calibration curve was constructed through the area under the curve (AUC) and results were expressed as μmol TE/g of dry sample. For total polyphenol content, ABTS and ORAC-FL assays, all samples were prepared in duplicate and each one of the preparations was measured in triplicate.

- Intracellular ROS determination

Intracellular ROS were measured on normal human colon fibroblast cells (CCD-18Co) and RAW264.7 mouse macrophage cells were obtained from American Type Culture Collection (ATCC, Manassas, VA, USA) [12]. Healthy CCD-18Co cells were used since this extract is aimed as a functional food for the healthy population. On the other hand, RAW264.7 murine leukemia cells were used as a classical cellular model for inflammation studies. Cells were grown using 75 cm^2^ cell culture flasks in DMEM containing glucose (4.5 g/L) and supplemented with a solution of penicillin-streptomycin (1%), L-glutamine (1%) and heat-inactivated FBS (10%). For cellular assays, CCD-18Co and RAW264.7 cells were seeded in 96-well plates (10,000 cells/well and 80,000 cells/well, respectively). All cell lines were incubated at 37 °C with 5% CO_2_, 100% RH (relative humidity) for 24 h, up to cell-confluence was achieved. Samples (extract and digest) were prepared in PBS 10 mM pH 7.4 (10 mg/mL of dry matter) and filtered by 22 µm. All determinations were performed in triplicate and in three different cell passages.

Previous to ROS determinations, cell viability was tested on CCD-18Co and RAW264.7 cells by MTT assay according to Fernández-Fernández et al. [3] description to determine the possible cytotoxic effect of samples (extract and digest). Intracellular ROS were measured using the fluorescent probe DCFH-DA. For the physiological ROS determination, different concentrations of the samples (1–1000 µg/mL for extract and 50–1000 µg/mL for digest) were added to each well (150 µL) for 24 h. After incubation, 2 µL of DCFH-DA (5 mg/mL in DMSO) were added to each well and plates were incubated for 30 min. Then, the supernatants were removed, cells were washed once with PBS and the same samples solutions (150 µL) were added to each well measuring fluorescence in a microplate reader at 485 nm and 528 nm of excitation and emission wavelengths, respectively. After fluorescence measurement, and to correct ROS values with cell viability, 20 µL of MTT reagent was added to each well [3]:

(1) $$\%\;\mathrm{ROS}=\;\frac{{\mathrm{Fluorescence}}_{\mathrm{sample}}}{{\mathrm{Absorbance}}_{\mathrm{MTT}\;\mathrm{sample}}}*\frac{{\mathrm{Absorbance}}_{\mathrm{MTT}\;C-}}{{\mathrm{Fluorescence}}_{C-}}*100$$

In addition, two different treatments for the induction of oxidative stress were studied on cells: (1) “Prevention”, in which cells were pre-treated with the extract or digest for 24 h, and after the probe incubation, only the oxidative agent (t-BOOH 1 mM) was added for 30 min. (2) “Prevention with co-administration”, where cells were also pre-treated with the extract or digest for 24 h, and after the probe incubation, t-BOOH and samples were co-administrated for 30 min. 

##### Anti-Inflammatory Capacity

Anti-inflammatory properties were evaluated on RAW264.7 mouse macrophages by measuring NO production when stimulating inflammation by LPS (1 µg/mL), as described by Benayad et al. [13]. Prevention assay consisted on measuring NO on cells supernatants when incubated with samples for 24 h and then stimulated with LPS for another 24 h. Prevention with co-administration assay consisted on measuring NO on cells supernatants incubated with the samples for 24 h and then stimulated with a mixture of samples + LPS for another 24 h. Studied samples were concentrations of 250–1000 µg/mL of extract and 10–1000 µg/mL of digest prepared in cell culture medium (150 µL per well) incubated for 24 h. After incubation, 100 µL of cells supernatants were transferred to another 96-well plate and added 100 µL of Griess reagent to each well. After a 15-min incubation at room temperature, absorbance was measured at 550 nm. Sodium nitrite was used for the standard curve in a range of 0 to 10 µg/mL. Negative and positive controls were also tested (non-treated cells and cells treated only with LPS, respectively). 

##### Inhibition of α-Glucosidase and α-Amylase Enzymatic Activity

The effect of the bioaccessible compounds in the metabolism of carbohydrates was determined by the inhibition of α-glucosidase and α-amylase enzymatic activities. For α-glucosidase inhibition assay [3], acarbose was used as the pharmaceutical of reference of carbohydrases inhibition capacity. Fluorescence measurements were displayed at 37 °C each minute for 30 min at 360 ± 40 nm and 460 ± 40 nm of excitation and emission wave lengths, respectively. Dose-response curves {% Inhibition vs. [Extract or Standard] (mg/mL)} were constructed to obtain IC_50_ values. 

α-Amylase inhibition assay was performed as reported by Li et al. [14]. Briefly, 35 U/mL of α-amylase human saliva stock solution (Sigma powder 160 U/mg) and 1% *w*/*v* starch stock solution were prepared in 20 mM sodium phosphate buffer at pH 6.9. Samples mixtures consisting of 50 μL of enzyme solution (35 U/mL), 50 μL of buffer and 100 μL of different concentrations of samples were incubated at 37 °C for 10 min in Eppendorf tubes. Then, 100 μL of starch solution (1%, *w*/*v*) were added and incubated at 37 °C for 10 min, then dinitrosalicylic acid (400 μL) was added to terminate the reaction. All tubes were heated in boiled water for 10 min, followed by cooling in a water bath at room temperature. Measurements were performed at 540 nm in a microplate reader (200 μL per well). Sample blanks consisted of mixtures of 100 μL of different concentrations of samples, 200 µL of buffer and 400 µL of dinitrosalicylic acid. Positive control consisted of 50 μL of enzyme solution (35 U/mL), 150 μL of buffer and 100 μL of starch solution (1%, *w*/*v*). The inhibition capacity was calculated as follows:

(2) $$\%\;\alpha -\mathrm{amylase}\;\mathrm{Inhibition}=\;\frac{A_{C+}-\left(A_{\mathrm{sample}}-{\;A}_{\mathrm{blank}}\right)}{A_{C+}}\;\times 100$$

where A_C+_, absorbance value for positive control, A_sample_, absorbance of the reaction mixture (sample, enzyme, starch and dinitrosalicylic acid) and A_blank_ the absorbance of sample with buffer and dinitrosalicylic acid.

##### Glucose Transport

Glucose is absorbed through SGLT 1 and GLUT 2 transporters in the small intestine, expressed on the apical side of the epithelial cells of the intestine. The Bioanalytical Techniques Unit (BAT, Instituto de Investigación en Ciencias de la Alimentación, Madrid, Spain) kindly provided normal rat small intestine epithelial cells (IEC-6). These healthy intestinal cells were used also as a model since this extract is aimed as a functional food for the healthy population. Glucose transport across IEC-6 cell monolayers was evaluated using transwell plates with polycarbonate inserts (Transwell^®^ inserts, 0.4 µm pore size, 1.1 cm^2^). IEC-6 cells were seeded on 12-well Transwell plates (6.9 × 10^4^ cells/cm^2^, 7.6 × 10^4^ cells/well). The cells were grown for 10 days in complete medium at the apical (500 µL) and basolateral (1500 µL) sides allowing cells to differentiate. The medium (DMEM with 4.5 g/L glucose + FBS 10% *v*/*v* + L-Glutamine 1% *v*/*v* + antibiotics 1% *v*/*v*) was changed every 2 days up to cells reached differentiation (10 days). Monolayers integrity was evaluated by transepithelial electrical resistance (TEER) using a Millicell-ERS device (Millipore, Zug, Switzerland), until TEER measurements were stable.

Differentiated cell monolayer and wells were gently washed with PBS 10 mM (pH 7.4) containing CaCl_2_ and KCl. PBS was aspirated and cells incubated for 30 min (37 °C, 5% CO_2_) with PBS, simulating fasting state. After incubation (30 min), PBS was aspirated from the apical and basolateral sides, replaced with 500 µL of sample mixtures and fresh PBS placed in the apical and basolateral side, respectively. Sampling was performed at different times (0, 10, 30, 45, 60, 90, 120 min) by removing 100 µL from the basolateral side and replacing the same volume with PBS.

Phloretin and phloridzin dihydrate were used as reference inhibitors of glucose transporters GLUT-2 and SGLT-1, respectively. In order to evaluate glucose absorption through cell monolayer, different concentrations of glucose (25 and 100 mM) and the digest (25, 50 and 100 µg/mL of dry sample) or reference inhibitor (0.1 mM phloretin and 0.3 mM phloridzin) were placed on the apical side. All treatments were performed in three different cell passages.

Glucose absorption was determined by measuring glucose concentration at different times in the basolateral side by glucose oxidase assay. Glucose concentration was calculated considering the amount of glucose in 100 µL of aliquot taken from the basolateral side plus the amount of glucose in the previous aliquot.

### 2.3. Statistical Analysis

All experiments were performed in triplicate. The statistical analysis was carried out by analysis of variance (ANOVA). Results were expressed as means ± standard deviation (SD) (*n* = 3). Besides, Tukey test was applied to determine significant differences between values (*p* < 0.05) using Infostat v. 2015 program. Different letters indicate significant differences when *p* < 0.05. In the case of glucose absorption experiments, LSD Fisher test was applied to determine significant differences between values (*p* < 0.1 and *p* < 0.05) using the same program.

## 3. Results and Discussions

### 3.1. Tannat Grape Skin Polyphenolic Composition

According to the UHPLC-MS/MS method developed, the *Vitis vinifera* cv Tannat polyphenol constituents of grape skin extract are presented in Table 1, where flavonoids dominated. Tannat grape skin extract contained a variety of polyphenols including phenolic acids (3-phenyllactic acid, caffeic acid, cis-aconitic acid, gallic acid, syringic acid, vanillic acid), phenolic alcohols (vanillyl alcohol) and flavonoids [7-Hydroxy-2-(4-hydroxyphenyl)-4-oxo-3,4-dihydro-2H-chromen-5-yl β-D-glucopyranoside, astragalin isomer 1, astragalin isomer 2, eriodictyol, isorhamnetin, myricetin, quercetin-3-galacturonide, quercetin, quercetin-3β-D-glucoside, naringenin]. Gallic acid was the main phenolic acid present in the extract. As to flavonoids negative ESI results, quercetin 3-galacturonide, quercetin, isorhamnetin, quercetin-3β-D-glucoside, and myricetin, in descendent order of content were the main flavonoids present in the extract. The content of individual anthocyanins in Tannat grape skin extract is also presented in Table 1 in positive ESI results. As expected, malvidin derivatives were the predominant compounds mostly followed by petunidin derivatives [1,15].

### 3.2. Bioaccessibility of Bioactive Compounds with Potential for Reducing the Risk or Treating Diabetes

#### Bioaccessibility of Antioxidants

The presence of phenolic compounds in the extract was confirmed by the TPC determination using the Folin-Ciocalteau method. TPC of the extract was 114.6 ± 10.5 mg GAE/g and after in vitro digestion, TPC significantly decreased (*p* < 0.05) to 11.9 ± 1.0 mg GAE/g. Data on TPC of the extract are in agreement with those reported by Beres et al. [17].

**Figure 1:**
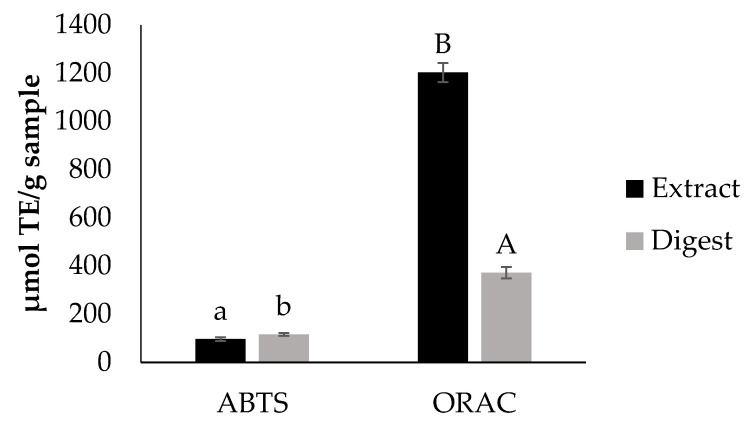
In vitro antioxidant capacity of the extract and the digest determined by ABTS and ORAC. Different letters indicate significant differences (Tukey test, *p* < 0.05) between values in the same assay.

Figure 1 shows the in vitro antioxidant capacity of the extract before and after the in vitro digestion. The overall antioxidant capacity obtained for the extract is in accordance to that reported by Beres et al. [17]. Results indicate that after the digestion process, the extract still possesses antioxidant properties. Nevertheless, this physiological event had an effect on the antioxidant capacity of the extract. Since the principle method of ABTS and ORAC is different, a different response was observed for the digest when using these two methodologies for antioxidant capacity evaluation. Data on the antioxidant capacity found with ORAC assay agreed with those on TPC suggesting the participation of these compounds in the observed property.

During digestion, transformation of polyphenols chemical structure and/or formation of complexes with food matrix macromolecules can occur resulting in the modification of bioactivities [17,18]. Particularly, anthocyanins have been found to be degraded by the transition from the gastric environment (acidic) to the intestinal environment (mild alkaline) and the effect of pancreatin and bile acids [19]. Moreover, flavonoids such as quercetin-3-O-glucoside [20] and myricetin [21], and phenolic acids such as gallic acid [22] have shown to degrade after in vitro digestion. Specifically in Tannat grape, after simulation of digestion, a decrease in anthocyanins and antioxidant capacity was observed, but the loss of antioxidant capacity was lower when compared with other red grape varieties [23].

The biological properties of the Tannat grape skin hydro-alcoholic-acid extract on cellular models are shown in Table S1. All studied extract concentrations caused a significant reduction (*p* < 0.05) of physiological intracellular ROS in a dose dependent manner in both cell lines. When oxidative stress was induced, the extract at concentrations higher than 100 µg/mL resulted as an effective inhibitor of induced ROS formation since ROS levels were significantly reduced (*p* < 0.05).

Results found by analysis of the digested extract on cellular models are shown in Table 2. As observed, the antioxidant properties of the extract remained bioaccessible after the in vitro digestion.

Table 2 shows data on intracellular ROS formation under physiological conditions (basal ROS) and induced by the oxidative agent t-BOOH (prevention and prevention with co-administration assays). The selection of the dose for treating the cells employed for studying the intracellular formation of ROS was based on cell viability data (Figure S1). Under physiological conditions, cyanidin chloride (20 µg/mL), used as antioxidant control, significantly reduced the intracellular formation of ROS in CCD-18Co (41.7 ± 10.6% ROS) and RAW264.7 cells (16.9 ± 3.1% ROS). In normal human intestinal cells, non-cytotoxic concentrations of the digest (100 and 500 μg/mL) under prevention with co-administration conditions, significantly reduced (*p* < 0.05) induced intracellular ROS. In RAW264.7 cells, the digest significantly reduced (*p* < 0.05) induced intracellular ROS under both studied conditions. These results agree with the data of individual polyphenols composition shown in Table 1, TPC and ORAC-FL found for the extract (Figure 1) and its digest. Nieto Fuentes [23] found out that wine by-products were important sources of bioavailable phenolic compounds using Caco-2 cells, which is in agreement with the current results for Tannat grape skin bioactivity after in vitro simulation of digestion.

### 3.3. Bioaccessibility of Anti-Inflammatory Compounds

NO generation by RAW264.7 macrophages was measured as an inflammation biomarker. Tannat grape skin hydro-alcoholic-acid extract significantly inhibited the formation of NO in a dose-dependent manner under prevention and prevention with co-administration conditions (Table S1).

The bioaccessible compounds of the extract (digest) significantly reduced (*p* < 0.05) NO formation in a dose-dependent manner under prevention and prevention with co-administration conditions (Table 2). Results seem to indicate a significantly higher effectivity (*p* < 0.05) of the digest under Prevention assay conditions showing at a concentration of 250 µg/mL a 59% NO formation decrease. The addition of the digest at 1000 µg/mL reduced NO formation to basal levels under prevention conditions (*p* > 0.05). However, a reduction of the same order of magnitude under prevention with co-administration conditions was observed using a digest dose of 1000 µg/mL.

Cyanidin chloride, one of the anthocyanins present in Tannat grape skin, presented significant differences (*p* < 0.05) compared to the positive control (LPS-induced inflammation) at concentration 20 µg/mL, which corresponds to total monomeric anthocyanins concentration in the extract at 1000 µg/mL. Cyanidin chloride exerted a bigger anti-inflammatory effect when tested in the prevention with co-administration assay (2.1 ± 0.3 µg/mL NO) reaching basal levels of NO production. As for red wines, Douro wines caftaric acid and malvidin-3-O-glucoside (59% of the determined compounds), were found to decrease NO production in LPS-induced RAW264.7 macrophages [24]. Anthocyanins, polyphenols composing Tannat grape skin, have already been reported for their anti-inflammatory effect by downregulating the expression of iNOS, COX-2, TNF-α, and IL-1β in murine BV2 microglial cells stimulated with LPS, as well as to inhibit LPS-induced pro-inflammatory context in the same type cells [25]. Malvidin-3-O-glucoside was one of the main anthocyanins present reported in the extract of the current work which may be the main responsible for this extract NO production inhibition in LPS-induced RAW264.7 macrophages.

Anti-inflammatory properties of grape pomace have been also reported in vivo. Red grape pomace methanolic extracts containing a great amount of anthocyanins suppressed chronic inflammation induced by LPS and galactosamine (GalN) in Sprague–Dawley rats when orally administered [26]. The extracts inhibited the activation of NF-κB by LPS/GalN stimulation in a dose-dependent manner. In vivo anti-inflammatory properties were also observed for Merlot and Petit Verdot red grape pomace [27,28].

The results of the present study seem to indicate that compounds capable of inhibiting the formation of NO, such as anthocyanins, which is of great importance in macrophages inflammatory response through inducible nitric oxide synthase (iNOS), remain bioaccesible after the digestion process of the extract. These compounds can exert an anti-inflammatory effect following different mechanisms of actions.

### 3.4. Bioaccessibility of Compounds with Potential to Modulate Glucose Metabolism

#### 3.4.1. Inhibitors of Enzymatic Activity of α-Glucosidase and α-Amylase

An important strategy for the post-prandial glycemic levels and diabetes care control is α-glucosidase and α-amylase inhibition by their role as the main digestive enzymes involved in the hydrolysis of starch [29]. Table 3 shows the effect of acarbose, the extract and the digest on the enzymatic activity of the enzymes involved in glucose metabolism.

The bioaccessibility of the inhibitors of both enzymes significantly decreased (*p* < 0.05) during the in vitro digestion of the extract (Table 3) resulting in IC_50_ values of 2945.7 ± 288.7 and 55,068.0 ± 1227.9 µg/mL for α-glucosidase and α-amylase inhibition, respectively. The degree of inhibition of the enzymatic activity depends on the chemical structure of the inhibitor. In the case of α-amylase, flavonoids hydroxyl groups (−OH) are essential for their inhibitory activity through the formation of hydrogen bonds between –OH and the active site, interacting phenolics with amino acids Asp^197^ and Glu^233^ side chains [29]. Glycosylation on flavonoids generally decreases the α-amylase inhibitory activity [29]. Tannins (proanthocyanidins and ellagitannins) from grape extracts showed inhibition of α-amylase enzymatic activity [30], being in accordance with the results of the present work. Anthocyanins delphinidin-3-glucoside and petunidin-3-glucoside present in the extract according to UHPLC-MS/MS results, were found to inhibit α-amylase and α-glucosidase activities, as well as a red grape extract anthocyanin fraction that showed an IC_50_ value of 589 ± 57 µg/mL for α-amylase inhibition and 80.9 ± 3.6 µg/mL for α-glucosidase inhibition [31]. Non-anthocyanins flavonoids, such as rutin and quercetin, are also potent inhibitors of α-glucosidase finding increased inhibition by the number of hydroxyl groups on the B-ring and glycosylation at both 3-OH and 5-OH [32], being quercetin present in the extract as well, according to UHPLC-MS/MS results.

#### 3.4.2. Modulation of Glucose Absorption

**Figure 2:**
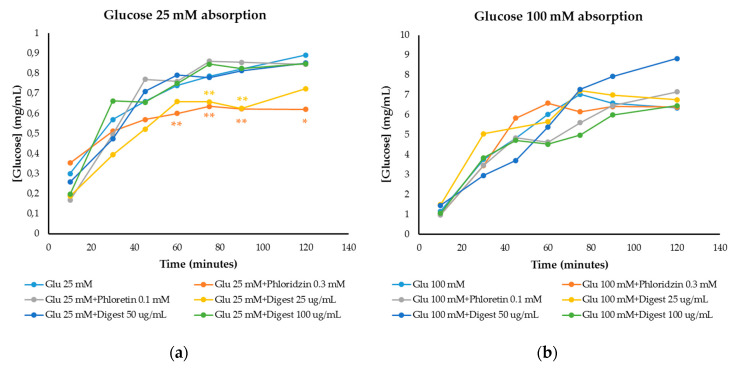
Glucose absorption in IEC-6 cells over time against reference inhibitors of glucose transporters (phloridzin and phloretin) and against the digestive simulation of the extract (digest) in the presence of (**a**) 25 mM glucose and (**b**) 100 mM glucose. Graphs of [Glucose] (mg/mL) vs. time (minutes). ANOVA analysis was performed by separated times using LSD Fisher test to state significant differences. *: *p* < 0.1; **: *p* < 0.05.

Glucose absorption results were obtained through the small intestine cell monolayer of IEC-6 cells (Figure 2), being able to determine the inhibition of intestinal glucose transporters (GLUT2 and SGLT1) by the digest. Phloretin and phloridzin were used as reference inhibitors of GLUT2 and SGLT1 glucose transporters, respectively. At 25 mM glucose concentration (Figure 2a), an intermediate/moderate glucose level in foods, inhibition of glucose absorption by phloridzin (0.3 mM) was observed compared to the control (25 mM glucose) at 60, 75 and 90 min (*p* < 0.05) and at 120 min (*p* < 0.1), while there was no inhibition of glucose permeability with phloretin (0.1 mM). At a concentration of 25 µg/mL, the digest presented the greatest inhibition, similar to that of phloridzin and with significant differences compared to the control at 60, 75 and 90 min (*p* < 0.05).

At 100 mM glucose (high glucose level in foods) (Figure 2b), a lower inhibition was observed with respect to the control (only 100 mM glucose). Phloridzin did not inhibit glucose absorption nor the digest at 25 µg/mL (*p* > 0.1 and *p* > 0.05). The main inhibition corresponded to the highest concentration of the digest (100 µg/mL) showing no significant differences (*p* > 0.1 and *p* > 0.05) compared to cells only treated with glucose 100 mM.

Some polyphenols such as piceid are absorbed through the glucose-dependent transporter SGLT1, which is involved in the transport of glycosylated flavonoids such as quercetin-3-O-glucoside, ending up as bioavailable compounds [23]. Thus, glycosylated flavonoids may compete with glucose for glucose transporters interaction, as occurs with the extract in the current work, whose composition determined by UHPLC-MS/MS showed this type of flavonoids.

Anthocyanins absorption through GLUT2 in Caco-2 cells has been demonstrated [33]. In vivo studies [34], have demonstrated that anthocyanins are absorbed and could be able to compete with glucose for glucose transporters. In addition, flavonols have shown to inhibit glucose and fructose transport through GLUT2 expressed in *Xenopus laevis* oocytes [35]. Furthermore, phenolic berry-extracts mainly composed by anthocyanins (approximately 60% *w*/*w*) were found to significantly inhibit both sodium-dependent (total uptake) and sodium-independent (facilitated uptake) glucose uptake on Caco-2 cells, as well as reducing significantly SGLT1 mRNA and GLUT2 mRNA expression [36].

Diabetes is characterized by hyperglycemia leading to its typical complications. Thus, controlling glucose levels after a meal is an important strategy for the prevention and/or treatment of this chronic disease. Inhibitors of glucose transport remained bioaccessible after digestion, suggesting the potential of the Tannat grape skin extract as an antidiabetic ingredient. To the best of our knowledge, this is the first time that Tannat grape skin extract is reported as a modulator of glucose absorption at the intestinal level.

## 4. Conclusions

UHPLC-MS/MS analysis of Tannat grape skin extract showed flavonoids, phenolic acids and phenolic alcohols as the main polyphenols. Our data support the in vitro bioaccessibility of Tannat grape skin extract compounds with potential to modulate key biochemical events involved in the pathogenesis of diabetes and to control the hyperglycemia due to this disease. Significant amounts of compounds able to inhibit intracellular induced formation of ROS in CCD-18Co (under prevention with co-administration conditions) and RAW264.7 cells (under prevention and prevention with co-administration conditions), LPS-induced inflammation of RAW264.7 macrophages and glucose transporters on small intestinal cells, remained bioaccessible after the in vitro digestion process. Concentrations of the digest of 100 and 250 µg/mL inhibited the formation of ROS and NO under induced conditions. To the best of our knowledge, this is the first study reporting the inhibition of glucose transport through small intestine monolayer by the bioaccessible fraction of the studied extract. The digest at 25 µg/mL significantly reduced (*p* < 0.05) glucose transport in IEC-6 cells under moderate glucose concentration conditions (25 mM). The extract resulted effective under prevention and prevention with co-administration conditions. In conclusion, our data support the potential of the intake of the Tannat extract hereby proposed for the reduction of the risk of diabetes or its treatment. Phenolic compounds seem to be contributors of the observed properties.

## Figures and Tables

**Table 1:** Data on identification of phenolic compounds composing the multifunctional extract from Tannat grape skin obtained under hydro-alcoholic-acid conditions analyzed by UHPLC-MS/MS.

**Negative ESI**				
**Compound ^1^**	**RT [min]**	**[M-H]^−^** **(*m/z*)**	**Fragments (*m/z*)**	**Extract ^2^**
3-Phenyllactic acid	10.6	165.0559	147.0455, 119.0504	0.0775
7-Hydroxy-2-(4-hydroxyphenyl)-4-oxo-3,4-dihydro-2H-chromen-5-yl β-D-glucopyranoside	11.8	433.1154	271.0607, 151.0041	0.0044
Astragalin isomer 1	11.1	447.0944	284.0334, 227.0356	0.0175
Astragalin isomer 2	11.2	447.0949	284.0334, 227.0356	0.0234
Caffeic acid	9.5	179.0354	135.0455	0.0172
cis-Aconitic acid	2.8	173.0094	129.0197, 85.0297	0.0575
Eriodictyol	13.6	287.0570	151.0041, 135.0456	0.0046
Gallic acid	3.7	169.0145	125.0247	0.2303
Isorhamnetin	14.9	315.0519	300.0283, 151.0037	0.1849
Myricetin	12.3	317.0310	178.9989, 151.0040	0.1094
Quercetin-3-galacturonide	10.7	477.0685	301.0361, 151.0039	0.4236
Quercetin	13.5	301.0361	151.0040, 107.0141	0.3677
Quercetin-3β-D-glucoside	10.7	463.0900	300.0282, 271.0254	0.1575
Syringic acid	11.2	197.0459	182.0225, 123.0091	0.0595
Vanillic acid	10.8	167.0352	152.0118, 123.0091	0.0450
Vanillyl alcohol	5.2	153.0561	138.0325, 123.0091	0.0336
Naringenin	14.8	271.0620	151.0041, 119.0505	0.0071
**Positive ESI**				
**Compound ^3^**	**RT [min]**	**[M]^+^ (*m/z*)**	**Fragments (*m/z*)**	**Extract ^2^**
Cyanidin 3-(6-O-acetylglucoside)	9.8	491.1184	287.0550	0.00001
Cyanidin-3-O-(6-p-coumaroyl) glucoside	10.9	595.1446	287.0550	0.00004
Cyanidin-3-pyranoside	8.5	449.1078	287.0550	0.00003
Delphinidin-3-(6-O-acetylglucoside)	9.2	507.1133	303.0500	0.00003
Delphinidin-3-O-(6-p-coumaroyl) glucoside	10.5	611.1395	303.0500	0.00015
Delphinidin-3-pyranoside	7.8	465.1027	303.0500	0.00010
Malvidin-3-(6-O-acetylglucoside)	10.4	535.1446	331.0800	0.00444
Malvidin-3-O-(6-p-coumaroyl) glucoside	11.5	639.1708	331.0800	0.00757
Malvidin-3-pyranoside	9.2	493.1340	331.0800	0.00886
Peonidin-3-(6-O-acetylglucoside)	10.4	505.1341	301.0700	0.00019
Peonidin-3-O-(6-p-coumaroyl) glucoside	11.5	609.1603	301.0700	0.00037
Peonidin-3-pyranoside	9.2	463.1235	301.0700	0.00033
Petunidin-3-(6-O-acetylglucoside)	9.9	521.1290	317.0700	0.00037
Petunidin-3-O-(6-p-coumaroyl) glucoside	11.0	625.1552	317.0700	0.00072
Petunidin-3-pyranoside	11.2	479.1184	317.0700	0.00051

^1^ Compound Discoverer 3.1 (mzCloud library, Advanced Mass Spectral Database); ^2^ Results normalized with TIC area (area/area TIC); ^3^ Ivanova et al. [16].

**Table 2:** Bioactive properties of bioaccessible compounds from Tannat grape skin hydro-alcoholic-acid extract (digest).

Digest	Assays		
(µg/mL)	Antioxidant Properties		
	**Intracellular ROS Formation in CCD-18Co Cells (%)**		
	**Physiological Conditions**	**Induced by t-BOOH (1 mM)**	
		**Prevention**	**Prevention with Co-Administration**
0	100.0 ± 6.3 ^a,A^	151.0 ± 12.7 ^a,b,B^	178.0 ± 7.0 ^b,B^
100	152.5 ± 39.2 ^b,A^	146.4 ± 30.5 ^a,b,A^	117.9 ± 30.0 ^a,A^
250	125.8 ± 13.7 ^a,b,A^	174.7 ± 57.2 ^b,A^	128.6 ± 21.2 ^a,A^
500	144.1 ± 10.4 ^b,A^	122.8 ± 13.8 ^b,A^	138.8 ± 32.0 ^a,A^
1000	135.1 ± 24.8 ^a,b,A^	192.4 ± 19.6 ^b,B^	185.3 ± 19.0 ^b,B^
	**Intracellular ROS Formation in RAW 264.7 Cells (%)**		
	**Physiological Conditions**	**Induced by t-BOOH (1 mM)**	
		**Prevention**	**Prevention with Co-Administration**
0	100.0 ± 8.3 ^a,A^	211.4 ± 44.4 ^b,B^	211.4 ± 44.4 ^b,B^
100	98.8 ± 20.0 ^a,A^	136.6 ± 15.8 ^a,B^	150.1 ± 39.3 ^a,B^
250	143.2 ± 39.1 ^b,A^	132.2 ± 10.9 ^a,A^	110.8 ± 21.4 ^a,A^
500	99.3 ± 24.3 ^a,A^	150.2 ± 21.1 ^a,B^	124.8 ± 16.6 ^a,A,B^
1000	116.3 ± 44.8 ^a,b,A^	99.4 ± 27.9 ^a,A^	129.9 ± 46.4 ^a,A^
**Anti-inflammatory Properties**			
**(µg/mL of NO Formation in RAW 264.7 Cells Induced by LPS 1 µg/mL)**			
	**Prevention**	**Prevention with Co-Administration**	
0	9.9 ± 0.8 ^c,A^	9.9 ± 0.8 ^c,A^	
100	5.3 ± 0.7 ^b,A^	5.6 ± 0.9 ^b,A^	
250	4.1 ± 0.9 ^a,A^	5.4 ± 1.0 ^b,B^	
500	3.3 ± 0.6 ^a,A^	4.9 ± 0.7 ^a,b,B^	
1000	3.1 ± 1.1 ^a,A^	4.0 ± 1.2 ^a,A^	

Results are expressed as mean values ± SD (*n* = 3). Different letters indicate significant differences (Tukey test, *p* < 0.05) between values in the same column (in lower case) or in the same row (capital letters). All determinations were performed in triplicate in three different cell passages.

**Table 3:** Bioaccessibility of inhibitors of carbohydrases in Tannat grape skin hydro-alcoholic-acid extract. IC_50_, half maximal inhibitory concentration.

Sample	α-Glucosidase Activity (IC_50_, μg/mL)	α-Amylase Activity (IC_50_, μg/mL)
Acarbose	4.0 ± 0.3 ^a^	34.1 ± 0.8 ^a^
Extract	888.5 ± 79.3 ^b^	1855.8 ± 21.3 ^b^
Digest	2945.7 ± 288.7 ^c^	55,068.0 ± 1227.9 ^c^

Results are expressed as mean values ± SD (*n* = 3). Sample solutions were prepared in triplicate and assayed in triplicate. Different letters denote significant differences (Tukey, *p* < 0.05) between values in the same column.

## Supplementary Materials

The following are available online at https://www.mdpi.com/2304-8158/9/11/1575/s1, Figure S1: Cell viability of IEC-6 (A), CCD-18Co and RAW264.7 (B) cells by MTT assay. Hydro-alcoholic-acid extract (EHAA) and its digestion (DEHAA) were tested. Negative control (C-), medium without FBS and DMSO 50%, was the positive control. Bars and error bars represent the mean values and standard deviation, respectively. Different letters state significant differences by Tukey test (*p* < 0.05). Table S1: Tannat grape skin hydro-alcoholic-acid extract bioactive properties on cellular models.
