# Supplementary material for: In Vitro Bioaccessibility of Extractable Compounds from Tannat Grape Skin Possessing Health Promoting Properties with Potential to Reduce the Risk of Diabetes
Source: Foods. 2020 Oct 30;9(11):1575. doi: 10.3390/foods9111575 (PMC7692155; doi:10.3390/foods9111575)
Supplement: Supplementary file 1 [file foods-09-01575-s001.pdf]

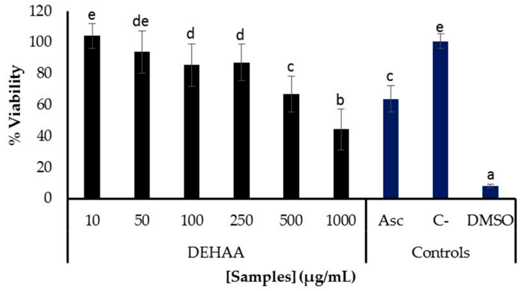

(**a**)

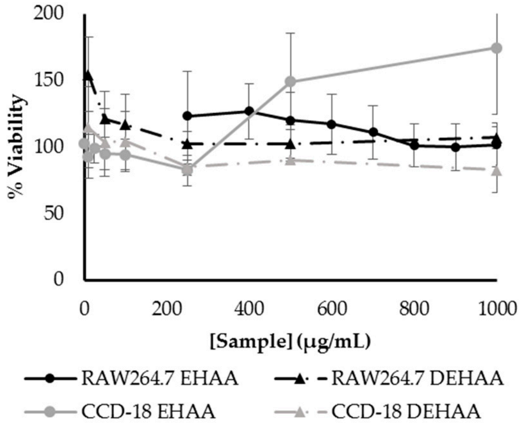

(**b**)

Figure S1. Cell viability of (**a**) IEC-6, (**b**) CCD-18Co and RAW264.7 cells by MTT assay. Hydro-alcoholic-acid extract (EHAA) and its digestion (DEHAA) were tested. Negative control (C-), medium without FBS and DMSO 50 %, was the positive control. Bars and error bars represent the mean values and standard deviation, respectively. Different letters state significant differences by Tukey test ($p<0.05$).

Table S1. Tannat grape skin hydro-alcoholic-acid extract bioactive properties on cellular models.

Extract (μg/mL)	Assays	
	Antioxidant properties	
	Intracellular ROS formation in CCD-18Co cells (%)	
	Physiological conditions	Induced by t-BOOH (1 mM)	
		Prevention	Prevention with co-administration
0	100.0±6.3^{cA}	151.0±12.7^{cB}	178.0±7.0^{cC}
100	41.5±8.4^{bA}	94.5±16.8^{abB}	115.8±11.9^{bC}
250	27.4±6.4^{aA}	78.8±13.1^{aB}	96.8±9.0^{bC}
500	24.6±8.6^{aA}	96.2±9.0^{abC}	65.5±16.2^{aB}
1000	24.4±7.7^{aA}	113.6±26.6^{bC}	59.7±16.3^{aB}
	Intracellular ROS formation in RAW 264.7 cells (%)		
	Physiological conditions	Induced by t-BOOH (1 mM)	
		Prevention	Prevention with co-administration
0	100.0±8.3^{cA}	211.4±44.4^{bB}	211.4±44.4^{bB}
100	38.9±8.3^{bA}	203.7±23.6^{abB}	59.0±11.2^{aA}
250	18.0±2.9^{aA}	167.7±20.0^{bC}	44.2±11.1^{aB}
500	13.0±2.7^{aA}	158.7±26.8^{bC}	38.3±12.3^{aB}
1000	14.3±3.8^{aA}	176.6±10.8^{bB}	23.6±1.6^{aA}
	Anti-inflammatory properties (μg/mL of NO formation in RAW 264.7 cells induced by LPS 1 μg/mL)		
	Prevention	Prevention with co-administration	
0	9.9±0.8^{dA}	9.9±0.8^{cA}	
250	8.3±0.7^{cA}	9.6±0.9^{cB}	
500	5.8±0.7^{bA}	7.6±0.2^{bB}	
800	2.9±0.5^{aA}	2.4±0.3^{aA}	
1000	2.4±0.4^{aA}	2.7±0.3^{aA}	

Results are expressed as mean values ± SD (n=3). Different letters indicate significant differences (Tukey test, $p<0.05$) between values in the same column (in lower case) or in the same row (capital letters). All determinations were performed in triplicate in three different cell passages.
